# GDF-15 as a proxy for epigenetic aging: associations with biological age markers, and physical function

**DOI:** 10.1007/s10522-024-10165-z

**Published:** 2024-12-07

**Authors:** Margalida Torrens-Mas, Cayetano Navas-Enamorado, Aina Galmes-Panades, Luis Masmiquel, Andrés Sanchez-Polo, Xavier Capo, Marta Gonzalez-Freire

**Affiliations:** 1 Translational Research in Aging and Longevity (TRIAL) Group, Health Research Institute of the Balearic Islands (IdISBa), 07120 Palma, Spain; 2 Grupo Multidisciplinar de Oncología Traslacional, Institut Universitari d´Investigació en Ciències de La Salut (IUNICS), University of the Balearic Islands, 07122 Palma, Spain; 3 Physical Activity and Sport Sciences Research Group (GICAFE), Institute for Educational Research and Innovation (IRIE), University of the Balearic Islands, 07122 Palma, Spain; 4 Consorcio CIBER, M.P. Fisiopatología de La Obesidad y Nutrición (CIBERObn), Instituto de Salud Carlos III (ISCIII), 28029 Madrid, Spain; 5 Vascular and Metabolic Pathologies Group, Health Research Institute of the Balearic Islands (IdISBa), 07120 Palma, Spain; 6 Research Group in Community Nutrition and Oxidative Stress, University of the Balearic Islands-Institut Universitari d´Investigació en Ciències de La Salut (IUNICS), 07122 Palma, Spain

**Keywords:** GDF-15, Aging, Biological aging, Epigenetic clocks, Physical performance, Inflammation

## Abstract

Growth differentiation factor 15 (GDF-15) has emerged as a significant biomarker of aging, linked to various physiological and pathological processes. This study investigates circulating GDF-15 levels in a cohort of healthy individuals from the Balearic Islands, exploring its associations with biological age markers, including multiple DNA methylation (DNAm) clocks, physical performance, and other age-related biomarkers. Seventy-two participants were assessed for general health, body composition, and physical function, with GDF-15 levels quantified using ELISA. Our results indicate that GDF-15 levels significantly increase with age, particularly in individuals over 60. Strong positive correlations were observed between GDF-15 levels and DNAm GrimAge, DNAm PhenoAge, Hannum, and Zhang clocks, suggesting that GDF-15 could serve as a proxy for epigenetic aging. Additionally, GDF-15 levels were linked to markers of impaired glycemic control, systemic inflammation, and physical decline, including decreased lung function and grip strength, especially in men. These findings highlight the use of GDF-15 as a biomarker for aging and age-related functional decline. Given that GDF-15 is easier to measure than DNA methylation, it has the potential to be more readily implemented in clinical settings for broader health assessment and management.

## Introduction

Growth differentiation factor 15 (GDF-15) is a stress-induced cytokine that belongs to the transforming growth factor beta (TGF-β) family (Candia et al. 2021). Circulating GDF-15 levels are relatively low under physiological conditions, although they rapidly increase in response to other cytokines, such as interleukin 1β or tumor necrosis factor alpha (TNFα) (Iglesias et al. 2023), tissue injury, or hypoxia, among others (Candia et al. 2021; Wischhusen et al. 2020). GDF-15 is believed to contribute to resolving inflammation and protect cells from apoptosis (Candia et al. 2021; Iglesias et al. 2023; Pence 2022). As its receptor has been found in the brainstem, evidence suggest that GDF-15 also regulates energy homeostasis through the control of appetite and body weight (Iglesias et al. 2023; Miyake et al. 2021).

Furthermore, GDF-15 levels have been positively associated with the aging process. In fact, Tanaka et al. (Tanaka et al. 2018) showed that this cytokine had the strongest positive correlation with age in humans, and several reports describe higher levels of GDF-15 in older individuals (Semba et al. 2020; Doerstling et al. 2018; Liu et al. 2020). Aging is characterized by a decline in physiological function and changes in body composition, being a major risk factor for a variety of chronic diseases. As such, GDF-15 is also associated with several age-related diseases, including cardiovascular disease (Echouffo-Tcheugui et al. 2021), cancer (Wischhusen et al. 2020), metabolic syndrome (Ho et al. 2023; Carballo-Casla et al. 2022), or diabetes (Ouyang et al. 2020; Merchant et al. 2023), among others (Candia et al. 2021; Iglesias et al. 2023). In addition, it has been proposed as a biomarker for the risk of death in patients with cardiovascular conditions and an accurate all-cause mortality marker (Candia et al. 2021; Iglesias et al. 2023; Nopp et al. 2021). GDF-15 has also been positively associated with deteriorated muscle function and sarcopenia (Semba et al. 2020; Kim et al. 2022, 2020; Nakajima et al. 2019; Lee et al. 2022), a highly prevalent condition among the elderly that increases the risk of frailty (Picca et al. 2020).

It is widely accepted that human aging may be influenced by epigenetic alterations (López-Otín et al. 2023). In this sense, age biomarkers based on DNA methylation have proven useful in predicting the risk of age-related diseases and mortality (Fransquet et al. 2019). Among several developed epigenetic clocks, DNAm GrimAge has shown a higher prediction capacity of mortality and morbidity risk (Lu et al. 2022). Notably, GDF-15 is one of the markers included for the calculation of this clock (Lu et al. 2019). Thus, understanding the interplay between GDF-15 and aging can be crucial for improving the assessment of and management of age-associated conditions.

For all this, the aim of this study was to characterize the changes in circulating GDF-15 levels with age in a population of healthy individuals from the Balearic Islands and investigate its potential associations with different epigenetic and biological clocks, physical performance and other age-related biomarkers.

## Methods

### Participant recruitment

The study included 72 participants from the Balearic Islands Study of Aging (BILSA Study) recruited from February 2022 to October 2022 in the Hospital Universitari Son Espases. All individuals were subjected to different physical tests and questionaries to evaluate their general health status and physical performance. The study was conducted according to the “World Medical Association Declaration of Helsinki” for research involving humans and was approved by the ethics committee of the Balearic Islands (Comitè d’Ètica de la Investigació de les Illes Balears, IB 4337/20 PI). All individuals were properly informed about the study and its risks and signed the informed consent.

The exclusion criteria included cognitive impairment, muscle or neuromuscular diseases, and a history of cancer within the previous ten years. Controlled diseases such as hypertension, diabetes, or hypercholesterolemia were included, as well as former smokers or light smokers. To avoid any increase in inflammatory markers in the blood that might have an impact on the outcomes, participants were also told not to exercise 24 h before to the study. All individuals were properly informed about the study and its risks and signed the informed consent.

Blood and buffy coat samples were obtained from each participant and stored at -80 °C at the Biobank Unit until use. Clinical data of the individuals included in this study include age, gender, smoking habit, comorbidities, and a general blood test in fasting conditions.

### Health questionnaires

Self-perceived health was assessed using a standardized questionnaire with a single-item measure, asking participants to rate their overall health on a five-point Likert scale (Excellent, Very good, Good, Fair, Poor) (ref) The data collection was conducted via [method], ensuring anonymity and confidentiality. Frailty status was evaluated using the Clinical Frailty Scale (CFS), a nine-point scale ranging from 1 (Very Fit) to 9 (Terminally Ill), based on clinical judgment and participant interviews (ref). Trained researchers assessed the CFS, considering factors such as physical activity, functional dependence, and the presence of comorbidities. Participants were categorized according to their frailty status.

### Physical performance tests

All individuals underwent a series of common physical performance tests, including the 4-m walking speed, grip strength, and the 5-times sit-to-stand test. A demonstration for every test was given to acquaint the participants with the process. For the 4-m walking speed, there were marks on the floor that served as the beginning and end marks, and each individual was asked to walk this distance at their usual and fast pace (Peel et al. 2013). Muscle strength was evaluated using a hand dynamometer (KERN MAP 80 K1, KERN, Germany), which was adjusted with known weights for appropriate fit and comfort. Participants were told to maintain a comfortable, still posture with their arms, and both hands were used to complete the test for a maximum of three tries. Then, the mean of each arm was recorded as the grip strength (Carson 2018). Finally, for the chair test, the participants were asked to rise five times from a seated position with their back against a chair without using their arms (Millor et al. 2013). They were told to move as quick as possible, without moving their arms and without stopping in between repeats. Each stand was counted aloud to keep the participants focused.

### Spirometry

A spirometer model Medi soft-5000 (Medi soft, Sorinnes, Belgium) was used for forced spirometry, and a disposable Lilly type transducer was used. The accuracy of the device was checked every day. Focusing on the lung function measures FEV1 (forced expiratory volume in the first second), FVC (forced vital capacity), and their ratio (FEV1/FVC), the spirometry parameters were measured as a percentage of the expected values (Catalin et al. 2023).

### Body composition

Body composition was analyzed for each participant with the InBody 770 bioelectrical impedance analysis (BIA) system (InBody Co. Ltd. In Korea). Prior to the exam, participants were given instructions to dress comfortably, not to wear any metal objects or electronic medical devices, to abstain from drinking at least two hours before the test and to fast for twelve hours. Subjects lined up with the back foot electrode while standing barefoot on the BIA equipment. They were told to maintain their arms away from their sides and to grip the hand electrodes until the exam was concluded.

### Determination of GDF-15 circulating levels

GDF-15 levels were measured using the Human GDF-15 Quantikine® ELISA Kit (#DGD150, R&D Systems) in plasma samples obtained from the 72 participants. Samples were diluted 1:4 with the Calibrator Diluent according to the manufacturer’s instructions and the protocol was followed as detailed in Torrens-Mas et al. (2022). All samples were run in duplicate.

### DNA methylation and epigenetic clock analysis

Using the DNeasy Blood & Tissue Kit (Qiagen, Hilden, Germany) and the Zymo EZ DNA Methylation Kit (Zymo Research, Irvine, CA), genomic DNA was isolated from blood samples and bisulfite converted in accordance with the manufacturer’s instructions. Using the internal controls included in the kit, the bisulfite conversion efficiency was assessed. The Illumina Infinium MethylationEPIC BeadChip array was used to assess the samples’ DNA methylation state (Illumina, San Diego, CA, USA). At the Clock Foundation UK, hybridization, cleaning, staining, and scanning of the arrays were carried out in accordance with the manufacturer’s instructions. Quality control (QC) steps included inspection of sample-dependent and sample-independent control probes, checking for sex mismatches, and filtering out poor-quality samples and probes. Probes with detection p-values > 0.01, non-CpG probes, and probes located on the sex chromosomes were excluded from the analysis. The specific epigenetic “clocks” chosen for use in this study were those derived by Horvath et al. (DNAm age and the Skin Blood clock) (Horvath 2013; Horvath et al. 2018), Hannum et al. (DNAm age H) (Hannum et al. 2013), Levine et al. (DNAm PhenoAge) (Levine et al. 2018), Zhang et al. (Zhang et al. 2019), and Lu et al. (DNAm age G or GrimAge) (Lu et al. 2019).

### Measurement of plasma metabolites

Liquid chromatography tandem mass spectrometry (LC–MS/MS) was used to quantify the metabolites found in plasma samples. Following the manufacturer’s instructions, the absoluteIDQ p180 kit (Biocrates Life Science AG, Innsbruck, Austria) was used to extract metabolites and measure their concentrations using a 6500 QTrap instrument (AB Sciex, Framingham, MA, USA) connected to an Agilent 1290 infinity UHPLC system (Agilent, Santa Clara, CA, USA). The analysis did not include metabolites that were detected below the limit of detection.

### Statistical analysis

Descriptive characteristics of the participants are reported as the mean ± the standard error of the mean (SEM) or as percentages. Distribution of all variables were examined through histograms and boxplots. Normality was checked with the Shapiro–Wilk test. Non-normally distributed continuous data were compared using the Kruskal–Wallis test and the Dunn test for pairwise comparison. The relationships between variables were studied using Spearman or Pearson correlations. Multivariate linear regression models were used to test the relationships and potential interactions between various independent variables (GDF-15, age, and gender) and the dependent variable of interest (body composition parameters and amino acids). All analyses and plots were performed using RStudio 2024.04.1 (R Foundation for Statistical Computing, Vienna, Austria).

## Results

### Study population

Table 1 shows a summary of the main characteristics of the studied population. Of the 72 participants, 37 (51%) were male and 35 (49%) were female. The average age was similar between men (48.7 years, range 23–79) and women (46.0 years, range 20–85). There were no significant gender differences in comorbidities such as hypertension, hypercholesterolemia, obesity, or asthma.

**Table 1:** Main characteristics of the participants of the study

Participants characteristics				
	Total	Male	Female	*p*
N	72	37	35	
Age (years)	47.4 ± 1.68	48.7 ± 2.32	46.0 ± 2.46	0.422
Comorbidities				
Hypertension	5 (6.94%)	3 (8.11%)	2 (5.71%)	0.527
Hypercholesterolemia	9 (12.0%)	6 (16.2%)	3 (8.57%)	0.268
Obesity	4 (5.56%)	3 (8.11%)	1 (2.86%)	0.328
Asthma	5 (6.94%)	3 (8.11%)	2 (5.71%)	0.527
Body composition				
BMI (kg/m^2^)	**24.7 ± 0.42**	**25.7 ± 0.53**	23.5 ± 0.61*	**0.007**
Body fat mass (kg)	16.8 ± 0.82	15.7 ± 1.08	17.9 ± 1.23	0.191
Soft lean mass (kg)	**51.2 ± 1.33**	**59.1 ± 1.38**	42.6 ± 1.13*	** < 0.001**
Fat free mass (kg)	**54.3 ± 1.40**	**62.6 ± 1.45**	45.2 ± 1.20*	** < 0.001**
Fat percentage (%)	**23.6 ± 1.00**	**19.8 ± 1.11**	27.7 ± 1.41*	** < 0.001**
Visceral fat area (cm^2^)	77.2 ± 4.41	71.1 ± 5.33	84.0 ± 7.06	0.225
Waist Hip Ratio	0.90 ± 0.01	0.90 ± 0.01	0.90 ± 0.01	0.895
50 kHz body phase angle	**5.73 ± 0.09**	**6.16 ± 0.10**	5.27 ± 0.09*	**< 0.001**
Physical performance				
4-m gait speed, usual pace (m/s)	3.40 ± 0.06	3.32 ± 0.08	3.47 ± 0.08	0.196
4-m gait speed, fast pace (m/s)	2.21 ± 0.03	2.12 ± 0.04	2.30 ± 0.05*	**0.009**
Chair test (s)	**8.68 ± 0.25**	**8.30 ± 0.31**	9.12 ± 0.38	0.071
Grip Strength (kg)	**32.1 ± 1.29**	**40.1 ± 1.09**	23.0 ± 1.12*	**< 0.001**
GDF-15 levels				
GDF-15 (pg/mL)	451 ± 25.1	464 ± 38.3	439 ± 32.4	0.648

Significant results are highlighted in bold

Student’s t-test: * statistical difference between men and women (p-value < 0.05)

Regarding body composition, a significant BMI difference of 2.2 points was observed between men and women (25.7 kg/m^2^ vs. 23.5 kg/m^2^, p = 0.007). Soft lean mass and fat-free mass were also higher in men (p < 0.001), while women showed an increase in fat percentage (p < 0.001). No differences were found in body fat mass (p = 0.19), visceral fat area (p = 0.23), or waist-hip ratio (o = 0.90). Finally, muscle quality, as measured by the 50 kHz phase angle, was higher in men than in women (p < 0.001).

In terms of physical performance, women showed a faster gait speed at a fast pace (2.30 m/s) compared to men (2.12 m/s; p = 0.009). Contrarily, men exhibited significantly higher grip strength (40.1 kg) than women (23.0 kg, p < 0.001). There were no differences in the gait speed at usual pace or in the chair test.

### GDF-15 levels increase with age and are associated with different proxies of biological age

**Fig. 1:**
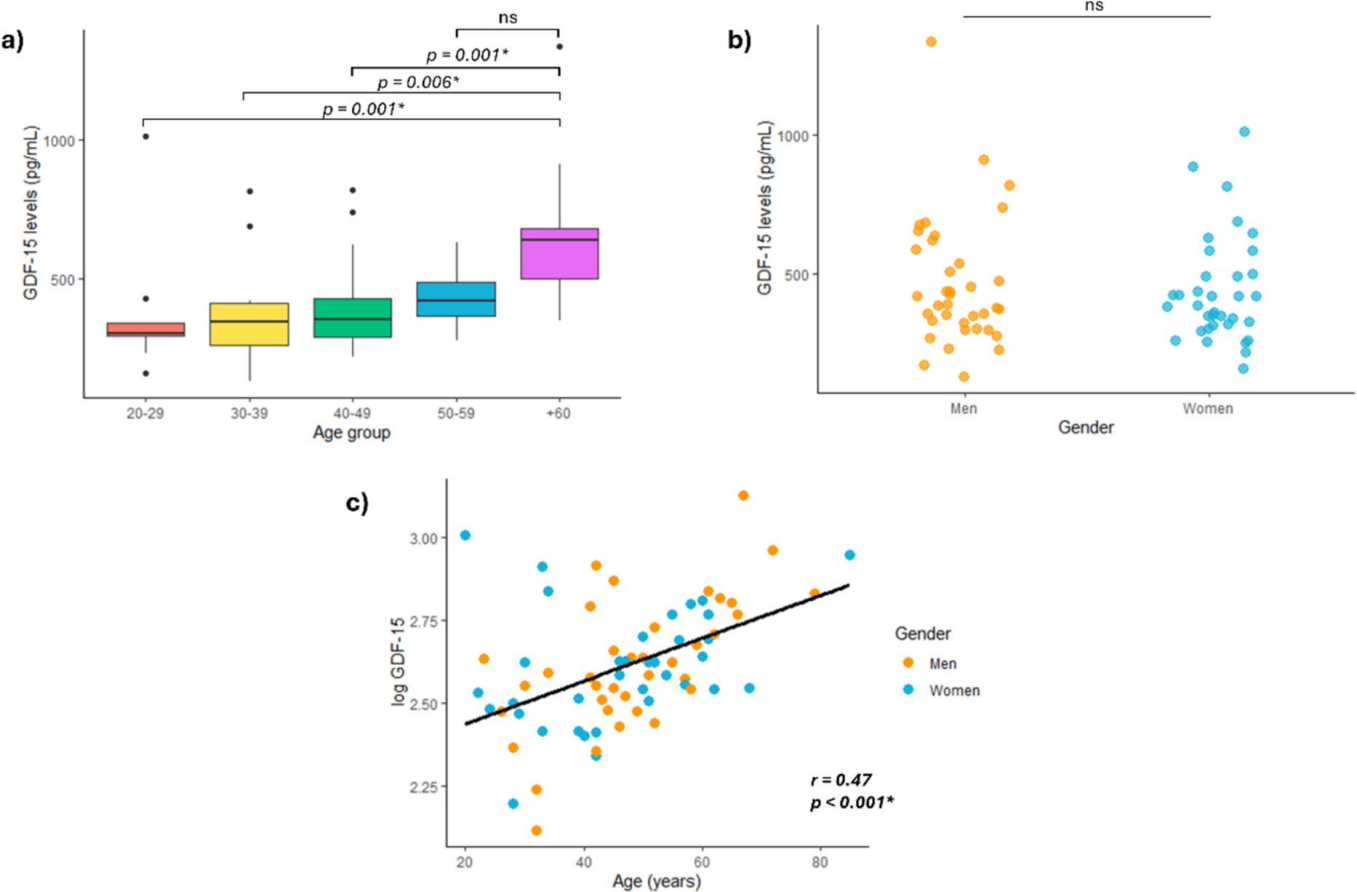
GDF-15 increase with age. **a** Boxplots showing the GDF-15 levels in different age groups: 20–29 years, 30–39 years, 40–49 years, 50–59 years, and 60 and above years. **b** GDF-15 levels in both genders. **c** Scatterplot showing the correlation of log GDF-15 and age in both genders. A linear regression line has been adjusted globally. ns = not significant

We first determined the levels of circulating GDF-15 in the serum samples of all participants, analyzing the differences between gender and various age groups. GDF-15 levels significantly increased with age, with individuals over 60 showing the highest levels (Fig. 1a). Particularly, the 60 and above group had greater levels of GDF-15 compared to the 20–29, 30–39, and 40–49 age groups, while no changes were found with the 50–59 age group. Additionally, there were no global differences between men and women (Fig. 1b), only in the group of individuals over 60 years men showed higher levels of GDF-15 than women (p = 0.04). A positive correlation was observed between age and GDF-15 levels (*r* = 0.475, p < 0.001; Fig. 1c). This correlation was stronger in men (*r* = 0.622, p < 0.001), while in women, it did not reach statistical significance (*r* = 0.319, p = 0.062).

**Fig. 2:**
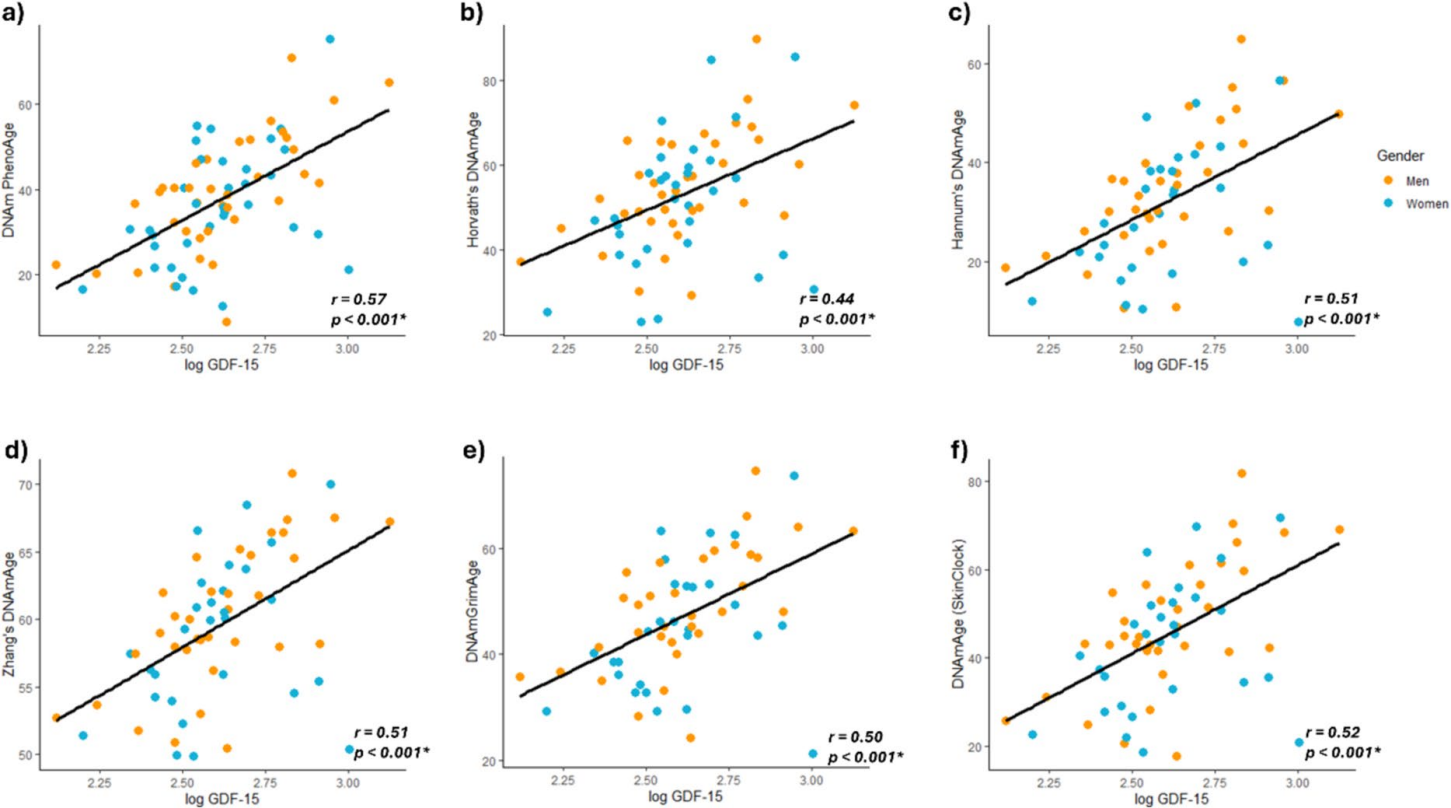
GDF-15 association with different epigenetic clocks. Scatterplots showing the correlation of log GDF-15 with **a** DNAm PhenoAge; b Horvath’s clock; **c** Hannum’s clock; **d** Zhang’s clock; **e** DNAmGrimAge estimation; and **f** Horvath’s skin and blood clock. A linear regression line has been adjusted globally

Next, we evaluated the association of circulating GDF-15 levels with different established epigenetic clocks. As shown in Fig. 2, GDF-15 was positively associated with the biological age estimated with different methods. Specifically, the stronger correlation was seen with the DNAm PhenoAge (*r* = 0.569, p < 0.001; Fig. 2a). The significance was maintained in both genders, although the correlation was higher in men (*r* = 0.681, p < 0.001) than in women (*r* = 0.44, p = 0.008). We also found a strong correlation between GDF-15 levels and the aging rate (*r* = 0.502, p < 0.001), calculated as the ratio between the PhenoAge and the chronological age (data not shown). The epigenetic clocks defined by Horvath, Hannun, and Zhang also showed a significant overall correlation with GDF-15 (*r* = 0.439, *r* = 0.508, and *r* = 0.514, respectively, p < 0.001; Fig. 2b, c, d). However, when analyzed by gender, this association was only seen in our male cohort (*r* = 0.565, *r* = 0.660, and *r* = 0.670, respectively, p < 0.001). Similarly, the GrimAge and the Skin Blood Clock also showed a significant global correlation with GDF-15 levels (*r* = 0.502 and *r* = 0.522, p < 0.001, respectively). Taking gender into account, these correlations were only found in men (*r* = 0.631 and *r* = 0.658), whereas in women they were not statistically significant (*r* = 0.349, p = 0.068, and *r* = 0.339, p = 0.078, respectively). No associations were found with other proxies of biological age, such as the extrinsic epigenetic age acceleration or the age acceleration residual. These results highlight the association between GDF-15 circulating levels and different measures of biological age, especially in men.

We further explored the relationship between GDF-15 levels and several epigenetically estimated biomarkers, such as telomere length, GDF-15, FGF-21, HGF, which have been previously associated with aging (Table 2). The levels of GDF-15 measured in our cohort and the estimated levels of GDF-15 based on DNA methylation showed a significant positive correlation (*r* = 0.534, p < 0.001). Additionally, a negative correlation was found between the levels of circulating GDF-15 and telomere length (*r* = −0.476, p < 0.001). Unfortunately, we were only able to measure telomere length in half of the participants of our cohort and did not observe the same trend. Serum GDF-15 also correlated with the estimated levels of FGF21 (*r* = 0.456, p < 0.001) and weakly with the estimated levels of HGF (*r* = 0.251, p = 0.035).

**Table 2:** Correlation coefficients between GDF-15 levels (log-transformed) and epigenetically estimated biomarkers

GDF-15 vs	*r*	*p*
Estimated GDF-15	**0.534**	< 0.001*
Estimated Telomere length	**−0.476**	< 0.001*
Estimated FGF21	**0.456**	< 0.001*
Estimated HGF	**0.251**	0.035*

Significant results are highlighted in bold

Pearson’s correlation: * significant correlation between variables (p-value < 0.05)

**Fig. 3:**
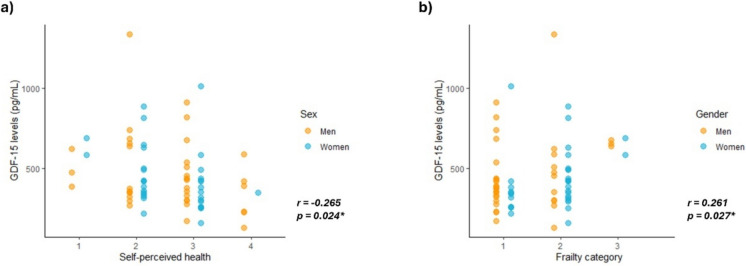
GDF-15 associated with self-perceived health and frailty. Scatterplots showing the correlation of GDF-15 levels with **a** Self-perceived health category, ranging in our cohort from 1 (fair) to 4 (excellent); and **b** Frailty category, ranging in our cohort from 1 (fit) to 3 (living with very mild frailty)

Finally, we also analyzed the association of GDF-15 levels with other proxies of biological age, including self-perceived health and the frailty category (Fig. 3). Interestingly, the self-perceived health category, rated from 1 (fair) to 4 (excellent), showed a slight but significant negative correlation with the levels of GDF-15 (*r* = −0.265, p = 0.024). In this line, the frailty category, ranging in our cohort from 1 (fit) to 4 (living with very mild frailty), was positively correlated with circulating GDF-15 levels (*r* = 0.261, p = 0.027).

### GDF-15 is related to pulmonary function and physical function tests

**Fig. 4:**
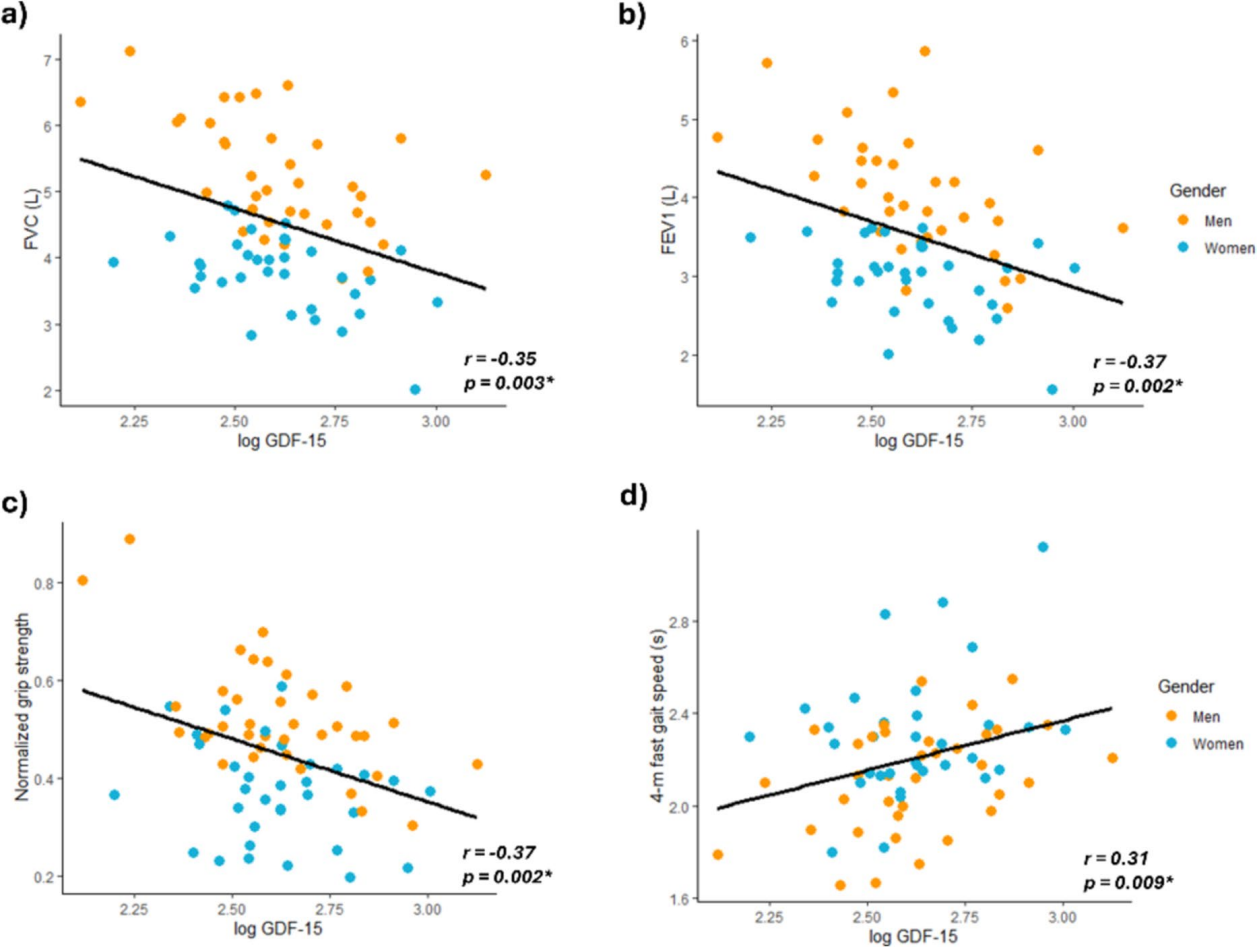
GDF-15 association with pulmonary and physical function tests. Scatterplots showing the correlation of log GDF-15 with **a** Forced vital capacity (FVC); **b** Forced expiratory volume (FEV1); **c** Grip strength normalized by body weight; d) 4-m gait speed at a fast pace. A linear regression line has been adjusted globally

To evaluate the connection of GDF-15 with functional parameters, we tested whether its levels are associated with pulmonary and physical function tests. A significant negative correlation was observed between GDF-15 and the forced vital capacity (*r* = -0.346, p = 0.003, Fig. 4a) and the forced expiratory volume (*r* = −0.367, p = 0.002, Fig. 4b). When analyzed by gender, these associations were slightly higher in men (*r* = −0.534, p < 0.001; *r* = −0.533, p < 0.001, respectively) than in women (*r* = −0.456, p = 0.006; *r* = −0.392, p = 022, respectively). We also found an overall negative correlation with the grip strength normalized by body weight (*r* = −0.366, p = 0.002, Fig. 4c), that was only significant in men (*r* = −0.608, p < 0.001).

Additionally, we found a positive correlation between the 4-m gait speed at a fast pace with the levels of GDF-15 (*r* = 0.308, p = 0.009, Fig. 4d). Again, this association was only observed in men when taking gender into account (*r* = 0.389, p = 0.017).

### GDF-15 levels are associated with body fat mass and muscle quality

Table 3 shows the correlations between the levels of GDF-15 and some measurements assessing body composition. We found a significant positive association between GDF-15 and several parameters related to the fat mass and cardiovascular risk, including the body mass index (*r* = 0.267, p = 0.024), the waist-hip ratio(*r* = 0.267, p = 0.024), the body fat mass (*r* = 0.349, p = 0.003), and the percentage of body fat (*r* = 0.302, p = 0.010), the visceral fat area (*r* = 0.341, p = 0.004), the fat mass index (*r* = 0.310, p = 0.008) and the obesity degree (*r* = 0.261, p = 0.028). Interestingly, these correlations were stronger in men, but were not observed in women. Other parameters of body composition, such as soft lean mass or fat free mass, showed no correlation with the levels of GDF-15. As parameters of body composition are strongly affected by gender and age, Supplemental Table 1 summarizes the lineal model adjusted for these variables. After adjusting for gender and age, GDF-15 levels do not show any significant associations with body composition, suggesting these variables are confounding factors. However, age is positively correlated with increased body fat mas and percentage, visceral fat area, and fat mass index, and negatively associated with fat free mass, soft lean mass, skeletal muscle index, and muscle quality. On the other hand, females show lower BMI, fat free mass, soft lean mass, skeletal muscle index, and muscle quality, but higher body fat percentage, visceral fat area, and fat mass index when compared to males.

**Table 3:** Correlation coefficients between GDF-15 levels (log-transformed) and parameters of body composition

	Total (N = 72)		Males (N = 37)		Females (N = 35)	
	R	*p*	r	*p*	r	*p*
BMI	**0.267**	0.024*	**0.386**	0.018*	0.152	0.392
Waist-hip ratio	**0.267**	0.024*	**0.429**	0.008*	0.090	0.614
Body fat mass (kg)	**0.349**	0.003*	**0.500**	0.002*	0.198	0.262
Body fat (%)	**0.302**	0.010*	**0.460**	0.004*	0.258	0.141
Visceral fat area (cm^2^)	**0.341**	0.004*	**0.512**	0.001*	0.203	0.250
Fat mass index	**0.310**	0.008*	**0.472**	0.003*	0.205	0.245
Obesity degree	**0.261**	0.028*	**0.362**	0.027*	0.161	0.363
50 kHz body phase angle	**-0.256**	0.031*	**-0.500**	0.001*	−0.128	0.472

Significant results are highlighted in bold

Pearson’s correlation: * significant correlation between variables (p-value < 0.05)

Finally, we observed a negative correlation between GDF-15 and muscle quality as estimated with InBody 50 kHz, which was greater in men (*r* = −0.500, p = 0.001) and non-significant in women (*r* = −0.128, p = 0.472). However, no association was found between GDF-15 and the skeletal muscle index or creatinine levels (shown in Supplemental Table 2). Interestingly, certain amino acids that contribute to muscle function also correlated with GDF-15 levels (Supplemental Table 2), particularly branched-chain amino acids (BCAAs, *r* = 0.256, p = 0.032), glutamate (*r* = 0.331, p = 0.009), lysine (*r* = 0.282, p = 0.028), proline (*r* = 0.286, p = 0.026), and taurine (*r* = 0.287, p = 0.025). These associations were stronger in men, except for taurine, which showed a strong correlation with GDF-15 only in women (*r* = 0.458, p = 0.014). After adjusting for gender and age, BCAAs, leucine, proline, and valine were the only amino acids remained significantly correlated with GDF-15 (Supplemental Table 3).

### GDF-15 is associated with circulating metabolic and inflammatory markers

Next, we analyzed the correlations between GDF-15 and parameters of glycemic control (Table 3). There was a significant association with the blood glucose levels (*r* = 0.311, p = 0.008), and when examined by gender, this correlation was only observed in men (*r* = 0.474, p = 0.003). Additionally, a significant correlation between glycosylated hemoglobin and GDF-15 levels was found in both genders (*r* = 0.536, p < 0.001 for men, and *r* = 0.371, p = 0.028, for women).

Lastly, as GDF-15 has been linked to systemic inflammation, we tested whether its levels are associated with circulating inflammatory markers. First, we observed a positive correlation between the levels of C-reactive protein (*r* = 0.243, p = 0.041) and the C-reactive protein-to-albumin ratio (*r* = 0.252, p = 0.034). Interestingly, these associations were not found in men, only in women (*r* = 0.357, p = 0.035; *r* = 0.356, p = 0.036, respectively). Other markers of inflammation, including D-dimer, plasminogen activity, urate, or the platelet-lymphocyte and neutrophil–lymphocyte ratios did not show any associations with serum GDF-15.

On the other hand, we calculated the kynurenine-tryptophan ratio in plasma to estimate the activity of 2,3-dioxygenase (IDO). This ratio revealed a positive correlation with the levels of GDF-15 (*r* = 0.235, p = 0.049), particularly in men (*r* = 0.385, p = 0.027). Lastly, phospholipase 2 activity in plasma, estimated by the ratio of lysophosphatidylcolines to total phosphatidylcolines, showed a significant negative correlation with GDF-15 only in men (*r* = -0.438, p = 0.011) (Table 4).

**Table 4:** Correlation coefficients between GDF-15 levels (log-transformed) and circulating inflammatory markers

	Total (N = 72)		Males (N = 37)		Females (N = 35)	
	r	*p*	r	*p*	r	*p*
Glucose (mg/dL)	**0.311**	0.008*	**0.474**	0.003*	0.024	0.892
HbA1c (mmol/mol)	**0.462**	< 0.001*	**0.536**	< 0.001*	**0.371**	0.028*
CRP (mg/dL)	**0.243**	0.041*	0.248	0.145	**0.357**	0.035*
CRP-albumin ratio	**0.252**	0.034*	0.249	0.143	**0.356**	0.036*
D-dimer (ng/mL)	0.147	0.248	0.226	0.205	-0.115	0.538
Plasminogen activity (%)	-0.006	0.973	-0.096	0.670	0.119	0.639
Urate (mg/dL)	0.095	0.429	0.083	0.627	0.117	0.504
Platelet-lymphocyte ratio	-0.121	0.313	-0.074	0.664	-0.157	0.369
Neutrophil–lymphocyte ratio	0.039	0.744	0.076	0.657	0.025	0.888
IDO activity (Kyn/Trp)	**0.253**	0.049*	**0.385**	0.027*	0.096	0.627
Phospholipase 2 activity	-0.179	0.167	**-0.438**	0.011*	-0.278	0.153

Significant results are highlighted in bold

Pearson’s correlation: * significant correlation between variables (p-value < 0.05). CRP: C-reactive protein

Finally, we also analyzed the correlation of GDF-15 levels with some inflammation-related markers that were predicted through DNA methylation patterns. Table 5 summarizes the main significant associations found in our cohort. We found an overall correlation between GDF-15 and the estimated count of naïve CD8 cells (*r* = −0.318, p = 0.008), the estimated levels of PAI-1 (*r* = 0.420, p < 0.001), TIMP-1 (*r* = 0.443, p < 0.001), CCL11 (*r* = 0.269, p = 0.034), and IL-6 (*r* = 0.289, p = 0.023). When taken gender into account, women only showed the correlation between GDF-15 and the estimated levels of PAI-1 (*r* = 0.451, p = 0.010), while in men GDF-15 levels were associated with the estimated number of naïve CD8 cells (*r* = −0.384, p = 0.021), the estimated levels of PAI-1 (*r* = 0.414, p = 0.011), and TIMP-1 (*r* = 0.613, p < 0.001).

**Table 5:** Correlation coefficients between GDF-15 levels (log-transformed) and estimated inflammatory markers

	**Total (N = 72)**		**Males (N = 37)**		**Females (N = 35)**	
	**r**	***p***	**r**	***p***	**r**	***p***
Estimated naive CD8	**-0.318**	0.008*	**-0.384**	0.021*	-0.238	0.190
Estimated PAI-1	**0.420**	< 0.001*	**0.416**	0.011*	**0.451**	0.010*
Estimated TIMP-1	**0.443**	< 0.001*	**0.613**	< 0.001*	0.242	0.181
Estimated CCL11	**0.269**	0.034*	0.259	0.139	0.285	0.142
Estimated IL-6	**0.289**	0.023*	0.274	0.117	0.310	0.108

Significant results are highlighted in bold

Pearson’s correlation: * significant correlation between variables (p-value < 0.05)

## Discussion

In this study, we explored the associations of serum GDF-15 levels with biological age, functional parameters, body composition, and inflammation-related markers to better understand its significance as a biomarker of the healthy aging process. Our results show that GDF-15 levels positively correlate with both chronological and biological age, as well as some inflammatory markers, while there was a negative correlation with pulmonary and physical function, as evidenced by the FVC and FEV1, grip strength, and gait speed. Notably, GDF-15 levels were not associated with body composition after adjusting for gender and age.

Previous research has shown that GDF-15 levels could serve as a biomarker for aging and some age-related conditions (Semba et al. 2020; Liu et al. 2021; Welsh et al. 2022). Consistent with this, our cohort’s plasma GDF-15 levels clearly increased with age. Furthermore, GDF-15 levels in our cohort fall within the range described for this cytokine (Welsh et al. 2022), corroborating the applicability and the validity of our measurements. In line with other reports, we only found a gender difference for GDF-15 levels when analyzing the 60 and above age group, in which men showed higher levels of this marker than women (Alcazar et al. 2021; Herpich et al. 2021; Mattia et al. 2023). This gender difference cannot be attributed to hormonal changes, any observed lifestyle differences, or the prevalence of age-related comorbidities, which is similar in both genders in our cohort. A greater rate of increase in plasma GDF-15 levels in men compared to woman throughout adulthood has been described (Alcazar et al. 2021), which could explain the gender difference in the older groups.

On the other hand, our results indicate that GDF-15 may also serve as a biomarker for biological aging and aging rate, as evidenced by its correlations with several biological clocks. Although these epigenetic clocks are currently considered the best predictors of biological age, a recent report showed a weak inter-correlation, suggesting that they capture different aspects of the aging process (Kuiper et al. 2023). Interestingly, the authors proposed that clocks trained on longitudinal or biophysiological data better reflect biological age, with DNAm GrimAge showing one of the best performances regarding frailty and mortality risk. In this line, we found the strongest correlation between GDF-15 and PhenoAge, which integrates clinical parameters to estimate biological age (Levine et al. 2018). Although these results need further validation, they highlight the relevance of GDF-15 in the context of aging research and its utility to complement markers of biological age.

Using blood methylation data, we also estimated the levels of some age biomarkers (Hillary and Marioni 2021) which we were unable to directly quantify in our cohort. As previously reported (Liu et al. 2021), GDF-15 levels negatively correlated telomere length. Telomere shortening has been described as a significant contributor to the aging process (Niu et al. 2024). GDF-15 was also associated with the estimated levels of FGF21. Previous studies have shown that these two cytokines are usually induced simultaneously and cooperate to regulate metabolism (Keipert and Ost 2021). Even though the levels of these markers are estimated, they further support the role of GDF-15 as an aging biomarker.

Additionally, our findings suggest that GDF-15 could serve as a marker of functional decline during the aging process, particularly in men, as evidenced by the negative correlation observed with lung and physical function measures. GDF-15 was found to be negatively correlated with FVC and FEV1, both indicators of lung function. Reduced FVC and FEV1 have been previously associated with higher GDF-15 levels, specifically in some diseases such as COPD (Husebø et al. 2017) or COVID-19 (Alserawan et al. 2021). Furthermore, GDF-15 also correlated with lower speed gait and grip strength. These functional parameters are widely used as indicators of muscle function and mobility, as well as predictors of frailty (Schrack et al. 2012; Vaishya et al. 2024). Thus, our results align with previous research showing that GDF-15 is associated with muscle wasting and increased frailty in the older population (Semba et al. 2020; Kim et al. 2022). Although after adjusting for confounding variables GDF-15 was not associated with muscle quality as measured with the 50 kHz body phase angle, we still observed a correlation of GDF-15 levels with lower grip strength and gait speed, as well as increased levels of BCAA in blood, which are involved in muscle metabolism and function (Dai et al. 2021; Caballero et al. 2023). Interestingly, when analyzed by gender, these associations disappeared in women, consistent with other reports (Herpich et al. 2021). Furthermore, we also found a positive correlation between GDF-15 levels and both self-perceived health and the frailty score. Altogether, these results suggest that GDF-15 could be used as a marker of an overall decline in lung and physical function during the aging process.

Finally, we found that GDF-15 levels correlated with impaired glycemia control and several inflammatory markers. GDF-15 has been linked before with glycosylated hemoglobin and poor blood glucose control in individuals with and without diabetes, suggesting a role in regulating metabolism (Kempf et al. 2012; Asrih et al. 2022). On the other hand, our results also support the link between GDF-15 and inflammation, as GDF-15 has been described as a cytokine induced by mitochondrial dysfunction and systemic inflammation (Moon et al. 2020), which are hallmarks of aging. Interestingly,

In conclusion, our study suggests GDF-15 as a useful biomarker for both healthy aging and functional decline, with gender-specific differences. Given that GDF-15 is easier to measure than DNA methylation, it has the potential to be more readily implemented in clinical settings. The significant associations with multiple physical performance parameters highlight the potential application of GDF-15 in age-related health assessment. However, the underlying mechanisms driving these associations and the role of GDF-15 in aging require further research, particularly through longitudinal studies.

## Data Availability

Data will be available under request to the corresponding author.

## References

[CR1] Alcazar J, Frandsen U, Prokhorova T, Kamper RS, Haddock B, Aagaard P, Suetta C (2021) Changes in systemic GDF15 across the adult lifespan and their impact on maximal muscle power: the copenhagen sarcopenia study. J Cachexia Sarcopenia Muscle 12:1418. 10.1002/JCSM.12823 34617415 10.1002/jcsm.12823 PMC8718085

[CR2] Alserawan L, Peñacoba P, Echevarría SEO, Castillo D, Ortiz E, Martínez-Martínez L, Naranjo EM, Domingo P, Castellví I, Juárez C et al (2021) Growth differentiation factor 15 (Gdf-15): a novel biomarker associated with poorer respiratory function in covid-19. Diagnostics. 10.3390/diagnostics11111998 34829345 10.3390/diagnostics11111998 PMC8625358

[CR3] Asrih M, Sinturel F, Dubos R, Guessous I, Pataky Z, Dibner C, Jornayvaz FR, Gariani K (2022) Sex-specific modulation of circulating growth differentiation factor-15 in patients with type 2 diabetes and/or obesity. Endocr Connect. 10.1530/EC-22-0054 35700236 10.1530/EC-22-0054 PMC9346339

[CR4] Caballero FF, Lana A, Struijk EA, Arias-Fernández L, Yévenes-Briones H, Cárdenas-Valladolid J, Salinero-Fort MÁ, Banegas JR, Rodríguez-Artalejo F, Lopez-Garcia E (2023) Prospective association between plasma amino acids and multimorbidity in older adults. J Gerontol Series A 78:637–644. 10.1093/GERONA/GLAC144 10.1093/gerona/glac144 35876753

[CR5] Carballo-Casla A, García-Esquinas E, Buño-Soto A, Struijk EA, López-García E, Rodríguez-Artalejo F, Ortolá R (2022) Metabolic syndrome and growth differentiation factor 15 in older adults. Geroscience 44:867–880. 10.1007/s11357-021-00370-w 33961185 10.1007/s11357-021-00370-w PMC9135918

[CR6] Carson RG (2018) Get a grip: individual variations in grip strength are a marker of brain health. Neurobiol Aging 71:189–222. 10.1016/j.neurobiolaging.2018.07.023 30172220 10.1016/j.neurobiolaging.2018.07.023

[CR7] Catalin R-E, Martin-Lujan F, Salamanca-Gonzalez P, Palleja-Millan M, Villalobos F, Santigosa-Ayala A, Pedret A, Valls-Zamora RM, Sola R (2023) Mediterranean diet and lung function in adults current smokers: a cross-sectional analysis in the MEDISTAR PROJECT. Nutrients 15:1272. 10.3390/nu15051272 36904270 10.3390/nu15051272 PMC10005310

[CR8] Dai M, Lin T, Yue J, Dai L (2021) Signatures and clinical significance of amino acid flux in sarcopenia: a systematic review and meta-analysis. Front Endocrinol (Lausanne) 12:725518. 10.3389/FENDO.2021.725518 34589057 10.3389/fendo.2021.725518 PMC8473793

[CR9] di Candia AM, de Avila DX, Moreira GR, Villacorta H, Maisel AS (2021) Growth differentiation factor-15, a novel systemic biomarker of oxidative stress, inflammation, and cellular aging: potential role in cardiovascular diseases. Am Heart J Plus 9:100046 38559370 10.1016/j.ahjo.2021.100046 PMC10978141

[CR10] Doerstling S, Hedberg P, Öhrvik J, Leppert J, Henriksen E (2018) Growth differentiation factor 15 in a community-based sample: age-dependent reference limits and prognostic impact. Ups J Med Sci 123:86. 10.1080/03009734.2018.1460427 29714603 10.1080/03009734.2018.1460427 PMC6055745

[CR11] Echouffo-Tcheugui JB, Daya N, Matsushita K, Wang D, Ndumele CE, Al Rifai M, Hoogeveen RC, Ballantyne CM, Selvin E (2021) Growth differentiation factor (GDF)-15 and cardiometabolic outcomes among older adults: the atherosclerosis risk in communities study. Clin Chem 67:653–661. 10.1093/clinchem/hvaa332 33582779 10.1093/clinchem/hvaa332 PMC8011530

[CR12] Fransquet PD, Wrigglesworth J, Woods RL, Ernst ME, Ryan J (2019) The epigenetic clock as a predictor of disease and mortality risk: a systematic review and meta-analysis. Clin Epigenetics 11:62. 10.1186/s13148-019-0656-7 30975202 10.1186/s13148-019-0656-7 PMC6458841

[CR13] Hannum G, Guinney J, Zhao L, Zhang L, Hughes G, Sadda S, Klotzle B, Bibikova M, Fan J-B, Gao Y et al (2013) Genome-wide methylation profiles reveal quantitative views of human aging rates. Mol Cell 49:359–367. 10.1016/j.molcel.2012.10.016 23177740 10.1016/j.molcel.2012.10.016 PMC3780611

[CR14] Herpich C, Franz K, Ost M, Otten L, Coleman V, Klaus S, Müller-Werdan U, Norman K (2021) Associations between serum GDF15 concentrations muscle mass, and strength show sex-specific differences in older hospital patients. Rejuvenation Res 24:14–19. 10.1089/REJ.2020.2308 32475214 10.1089/rej.2020.2308

[CR15] Hillary RF, Marioni RE (2021) MethylDetectR: a software for methylation-based health profiling. Wellcome Open Res 5:283. 10.12688/wellcomeopenres.16458.2 33969230 10.12688/wellcomeopenres.16458.1 PMC8080939

[CR16] Ho LC, Wu HT, Hung HC, Chou HW, Cheng KP, Lin CH, Wang CC, Ou HY (2023) Growth differentiation factor-15 is independently associated with metabolic syndrome and hyperglycemia in non-elderly subjects. BioFactors 49:119–126. 10.1002/biof.1871 35686301 10.1002/biof.1871

[CR17] Horvath S (2013) DNA methylation age of human tissues and cell types. Genome Biol 14:R115. 10.1186/gb-2013-14-10-r115 24138928 10.1186/gb-2013-14-10-r115 PMC4015143

[CR18] Horvath S, Oshima J, Martin GM, Lu AT, Quach A, Cohen H, Felton S, Matsuyama M, Lowe D, Kabacik S et al (2018) Epigenetic clock for skin and blood cells applied to hutchinson gilford progeria syndrome and Ex Vivo studies. Aging 10:1758–1775. 10.18632/aging.101508 30048243 10.18632/aging.101508 PMC6075434

[CR19] Husebø GR, Grønseth R, Lerner L, Gyuris J, Hardie JA, Bakke PS, Eagan TM (2017) Growth differentiation factor-15 is a predictor of important disease outcomes in patients with COPD. Eur Respir J 49:1601298. 10.1183/13993003.01298-2016 28298399 10.1183/13993003.01298-2016

[CR20] Iglesias P, Silvestre RA, Díez JJ (2023) Growth differentiation factor 15 (GDF-15) in endocrinology. Endocrine 81:419 37129758 10.1007/s12020-023-03377-9

[CR21] Keipert S, Ost M (2021) Stress-induced FGF21 and GDF15 in obesity and obesity resistance. Trends Endocrinol Metab 32:904–915. 10.1016/J.TEM.2021.08.008 34526227 10.1016/j.tem.2021.08.008

[CR22] Kempf T, Guba-Quint A, Torgerson J, Magnone MC, Haefliger C, Bobadilla M, Wollert KC (2012) Growth differentiation factor 15 predicts future insulin resistance and impaired glucose control in obese nondiabetic individuals: results from the XENDOS Trial. Eur J Endocrinol 167:671–678. 10.1530/EJE-12-0466) 22918303 10.1530/EJE-12-0466

[CR23] Kim H, Kim KM, Kang MJ, Lim S (2020) Growth differentiation factor-15 as a biomarker for sarcopenia in aging humans and mice. Exp Gerontol. 10.1016/j.exger.2020.111115 33069782 10.1016/j.exger.2020.111115

[CR24] Kim M, Walston JD, Won CW (2022) Associations between elevated growth differentiation factor-15 and sarcopenia among community-dwelling older adults. J Gerontol Series A Biol Sci Med Sci 77:770–780. 10.1093/gerona/glab201 10.1093/gerona/glab201 34255062

[CR25] Kuiper LM, Polinder-Bos HA, Bizzarri D, Vojinovic D, Vallerga CL, Beekman M, Dollé MET, Ghanbari M, Voortman T, Reinders MJT et al (2023) Epigenetic and metabolomic biomarkers for biological age: a comparative analysis of mortality and frailty risk. J Gerontol Series A 78:1753–1762. 10.1093/GERONA/GLAD137 10.1093/gerona/glad137 PMC10562890 37303208

[CR26] Lee SH, Lee JY, Lim KH, Lee YS, Koh JM (2022) Associations between plasma growth and differentiation factor-15 with aging phenotypes in muscle, adipose tissue, and bone. Calcif Tissue Int 110:236–243. 10.1007/s00223-021-00912-6 34499185 10.1007/s00223-021-00912-6

[CR27] Levine ME, Lu AT, Quach A, Chen BH, Assimes TL, Bandinelli S, Hou L, Baccarelli AA, Stewart JD, Li Y et al (2018) An epigenetic biomarker of aging for lifespan and healthspan. Aging 10:573–591. 10.18632/aging.101414 29676998 10.18632/aging.101414 PMC5940111

[CR28] Liu H, Huang Y, Lyu Y, Dai W, Tong Y, Li Y (2020) GDF-15 as a biomarker of aging. Exp Gerontol. 10.21203/rs.3.rs-30717/v133279668

[CR29] Liu H, Huang Y, Lyu Y, Dai W, Tong Y, Li Y (2021) GDF15 as a biomarker of ageing. Exp Gerontol 146:111228. 10.1016/J.EXGER.2021.111228 33421539 10.1016/j.exger.2021.111228

[CR30] López-Otín C, Blasco MA, Partridge L, Serrano M, Kroemer G (2023) Hallmarks of aging: an expanding universe. Cell 186:243–278. 10.1016/j.cell.2022.11.001 36599349 10.1016/j.cell.2022.11.001

[CR31] Lu AT, Quach A, Wilson JG, Reiner AP, Aviv A, Raj K, Hou L, Baccarelli AA, Li Y, Stewart JD et al (2019) DNA methylation grimage strongly predicts lifespan and healthspan. Aging 11:303–327. 10.18632/aging.101684 30669119 10.18632/aging.101684 PMC6366976

[CR32] Lu AT, Binder AM, Zhang J, Yan Q, Reiner AP, Cox SR, Corley J, Harris SE, Kuo P-L, Moore AZ et al (2022) DNA methylation GrimAge version 2. Aging. 10.18632/aging.204434 36516495 10.18632/aging.204434 PMC9792204

[CR33] Mattia L, Gossiel F, Walsh JS, Eastell R (2023) Effect of age and gender on serum growth differentiation factor 15 and its relationship to bone density and bone turnover. Bone Rep. 10.1016/j.bonr.2023.101676 37090856 10.1016/j.bonr.2023.101676 PMC10113774

[CR34] Merchant RA, Chan YH, Duque G (2023) GDF-15 Is associated with poor physical function in prefrail older adults with diabetes. J Diabetes Res. 10.1155/2023/2519128 37152099 10.1155/2023/2519128 PMC10162869

[CR35] Millor N, Lecumberri P, Gómez M, Martínez-Ramírez A, Izquierdo M (2013) An evaluation of the 30-s chair stand test in older adults: frailty detection based on kinematic parameters from a single inertial UNIT. J Neuroeng Rehabil 10:86. 10.1186/1743-0003-10-86 24059755 10.1186/1743-0003-10-86 PMC3735415

[CR36] Miyake M, Zhang J, Yasue A, Hisanaga S, Tsugawa K, Sakaue H, Oyadomari M, Kiyonari H, Oyadomari S (2021) Integrated stress response regulates GDF15 secretion from adipocytes, preferentially suppresses appetite for a high-fat diet and improves obesity. iScience 24:103448 34877504 10.1016/j.isci.2021.103448 PMC8633987

[CR37] Moon JS, Goeminne LJE, Kim JT, Tian JW, Kim SH, Nga HT, Kang SG, Kang BE, Byun JS, Lee YS et al (2020) Growth differentiation factor 15 protects against the aging-mediated systemic inflammatory response in humans and mice. Aging Cell 19:e13195. 10.1111/ACEL.13195 32691494 10.1111/acel.13195 PMC7431835

[CR38] Nakajima T, Shibasaki I, Sawaguchi T, Haruyama A, Kaneda H, Nakajima T, Hasegawa T, Arikawa T, Obi S, Sakuma M et al (2019) Growth differentiation factor-15 (GDF-15) is a biomarker of muscle wasting and renal dysfunction in preoperative cardiovascular surgery patients. J Clin Med. 10.3390/jcm8101576 31581569 10.3390/jcm8101576 PMC6832285

[CR39] Niu B, Wu JX, Huang XL, Lei SF, Deng FY (2024) Telomere length is a driving hallmark for aging-related biochemical hallmarks: evidence from the shared genetic effect and causal inference. J Gerontol Series A. 10.1093/GERONA/GLAD275 10.1093/gerona/glad275 38134301

[CR40] Nopp S, Königsbrügge O, Kraemmer D, Pabinger I, Ay C (2021) Growth differentiation factor-15 predicts major adverse cardiac events and all-cause mortality in patients with atrial fibrillation. Eur J Intern Med 88:35–42. 10.1016/j.ejim.2021.02.011 33706979 10.1016/j.ejim.2021.02.011

[CR41] Ouyang J, Isnard S, Lin J, Fombuena B, Peng X, Chen Y, Routy JP (2020) GDF-15 as a weight watcher for diabetic and non-diabetic people treated with metformin. Front Endocrinol (Lausanne). 10.3389/fendo.2020.581839 33312159 10.3389/fendo.2020.581839 PMC7708317

[CR42] Peel NM, Kuys SS, Klein K (2013) Gait speed as a measure in geriatric assessment in clinical settings: a systematic review. J Gerontol Series A 68:39–46. 10.1093/gerona/gls174 10.1093/gerona/gls174 22923430

[CR43] Pence BD (2022) Growth differentiation factor-15 in immunity and aging. Front Aging. 10.3389/fragi.2022.837575 35821815 10.3389/fragi.2022.837575 PMC9261309

[CR44] Picca A, Calvani R, Cesari M, Landi F, Bernabei R, Coelho-Júnior HJ, Marzetti E (2020) Biomarkers of physical frailty and sarcopenia: coming up to the place? Int J Mol Sci 21:1–16. 10.3390/ijms21165635 10.3390/ijms21165635 PMC7460617 32781619

[CR45] Schrack JA, Simonsick EM, Chaves PHM, Ferrucci L (2012) The role of energetic cost in the age-related slowing of gait speed. J Am Geriatr Soc 60:1811–1816. 10.1111/j.1532-5415.2012.04153.x 23035640 10.1111/j.1532-5415.2012.04153.x PMC3470763

[CR46] Semba RD, Gonzalez-Freire M, Tanaka T, Biancotto A, Zhang P, Shardell M, Moaddel R, Ferrucci L (2020) Elevated plasma growth and differentiation factor 15 Is associated with slower gait speed and lower physical performance in healthy community-dwelling adults. J Gerontol Series A Biol Sci Med Sci 75:175–180. 10.1093/gerona/glz071 10.1093/gerona/glz071 PMC6909888 30874790

[CR47] Tanaka T, Biancotto A, Moaddel R, Moore AZ, Gonzalez-Freire M, Aon MA, Candia J, Zhang P, Cheung F, Fantoni G et al (2018) Plasma proteomic signature of age in healthy humans. Aging Cell 17:1–13. 10.1111/acel.12799 10.1111/acel.12799 PMC6156492 29992704

[CR48] Torrens-Mas M, Perelló-Reus CM, Trias-Ferrer N, Ibargüen-González L, Crespí C, Galmes-Panades AM, Navas-Enamorado C, Sanchez-Polo A, Piérola-Lopetegui J, Masmiquel L et al (2022) GDF15 and ACE2 Stratify COVID-19 patients according to severity while ACE2 mutations increase infection susceptibility. Front Cell Infect Microbiol. 10.3389/fcimb.2022.942951 35937703 10.3389/fcimb.2022.942951 PMC9355674

[CR49] Vaishya R, Misra A, Vaish A, Ursino N, D’Ambrosi R (2024) Hand grip strength as a proposed new vital sign of health: a narrative review of evidences. J Health Popul Nutr 43:7. 10.1186/s41043-024-00500-y 38195493 10.1186/s41043-024-00500-y PMC10777545

[CR50] Welsh P, Kimenai DM, Marioni RE, Hayward C, Campbell A, Porteous D, Mills NL, O’Rahilly S, Sattar N (2022) Reference ranges for GDF-15, and risk factors associated with GDF-15, in a large general population cohort. Clin Chem Lab Med 60:1820–1829. 10.1515/cclm-2022-0135 35976089 10.1515/cclm-2022-0135 PMC9524804

[CR51] Wischhusen J, Melero I, Fridman WH (2020) Growth/differentiation factor-15 (GDF-15): from biomarker to novel targetable immune checkpoint. Front Immunol. 10.3389/fimmu.2020.00951 32508832 10.3389/fimmu.2020.00951 PMC7248355

[CR52] Zhang Q, Vallerga CL, Walker RM, Lin T, Henders AK, Montgomery GW, He J, Fan D, Fowdar J, Kennedy M et al (2019) Improved precision of epigenetic clock estimates across tissues and its implication for biological ageing. Genome Med 11:54. 10.1186/s13073-019-0667-1 31443728 10.1186/s13073-019-0667-1 PMC6708158

